# An effective detection method for wheat mold based on ultra weak luminescence

**DOI:** 10.1038/s41598-022-14344-1

**Published:** 2022-06-21

**Authors:** Gong Yue-hong, Yang Tie-jun, Liang Yi-tao, Ge Hong-yi, Chen Liang, Gao Hui, Shen Er-bo

**Affiliations:** 1 Key Laboratory of Grain Information Processing and Control, Henan University of Technology, Ministry of Education, Zhengzhou, China; 2 Henan Key Laboratory of Grain Photoelectric Detection and Control, Henan University of Technology, Zhengzhou, China; 3 School of Information Science and Engineering, Henan University of Technology, Zhengzhou, China; 4 School of Artificial Intelligence and Big Data, Henan University of Technology, Zhengzhou, China; 5 College of Biological Engineering, Henan University of Technology, Zhengzhou, China; 6 School of Mechanical and Electrical Engineering, Henan University of Technology, Zhengzhou, China

**Keywords:** Biotechnology, Optics and photonics

## Abstract

It is widely known that mold is one of important indices in assessing the quality of stored wheat. First, mold will decrease the quality of wheat kernels; the wheat kernels infected by mold can produce secondary metabolites, such as aflatoxins, ochratoxin A, zearalenone, fumonisins and so on. Second, the mycotoxins metabolized by mycetes are extremely harmful to humans; once the food or feed is made of by those wheat kernels infected by mold, it will cause serious health problems on human beings as well as animals. Therefore, the effective and accurate detection of wheat mold is vitally important to evaluate the storage and subsequent processing quality of wheat kernels. However, traditional methods for detecting wheat mold mainly rely on biochemical methods, which always involve complex and long pretreatment processes, and waste part of wheat samples for each detection. In view of this, this paper proposes a type of eco-friendly and nondestructive wheat mold detection method based on ultra weak luminescence. The specific implementation process is as follows: firstly, ultra weak luminescence signals of the healthy and the moldy wheat subsamples are measured by a photon analyzer; secondly, the approximate entropy and multiscale approximate entropy are introduced as the main classification features separately; finally, the detection model has been established based on the support vector machine in order to classify two types of wheat subsamples. The receiver operating characteristic curve of the newly established detection model shows that the highest classification accuracy rate can reach 93.1%, which illustrates that our proposed detection model is feasible and promising for detecting wheat mold.

## Introduction

Wheat, as a type of global grain, is one of the staple foods that human beings and animals rely on. The history of wheat cultivation can be traced back to ten thousand years ago, and wheat has become the second most cultivated crop in the world due to its high productivity and strong adaptability^1^. However, when a suitable surrounding moisture and temperature is available, microorganisms will make great contributions to trigger wheat mold phenomenon, thus decreasing the quality and quantity of storage wheat^2^. It is reported that the average loss of storage wheat caused by mold nearly takes up 2.1% of the total wheat yields annually in China^3^. In addition, the health of human beings will be extremely threatened once certain edible food is made of moldy wheat as raw materials^4^. The moldy wheat kernels will carry a wide variety of mold, such as *aspergillus flavus*, *aspergillus candidus*, *aspergillus glaucus*, *aspergillus nidulans*, *aspergillus pali*, *aspergillus versicolor*, *aspergillus terreus*, *aspergillus fumigatus*, *aspergillus niger*, and so on. Moreover, Many mycotoxins are metabolized by these molds, such as aflatoxin B1, aflatoxin B2, aflatoxin G1, aflatoxin G2, and so on, among which aflatoxin B1 (AFB1) is the most striking contaminant and has the strongest carcinogenicity^5^. The AFB1 is prone to cause a series of illnesses, such as retarded growth, immune suppression, human or animal death, and so on^6^. Therefore, research of an effective and nondestructive technique in detecting AFB1 for stored wheat is of necessity to ensure the security of human beings and animals.

Due to the low contents of fungaltoxin, conventional AFB1 detection methods are mainly involved in biochemical methods, such as fluorescence analysis method, determination of microbial activity method, molecular biology method, and so on^7,8^. Although above-mentioned methods have achieved fine-grained detection degree for wheat mold, all of them are time-consuming, high cost and involved in long pretreatments, which are difficult to meet the requirements of rapid on-site detection.

The study of biological photons was traced back to 1923 when the Russian biologist Gurwitsch used biological detectors to test the roots of onions and found a special phenomenon: onion cells were able to emit ultraviolet light that stimulated other cells to accelerate cell divisions^9^. In 1955, the Italian scientist Colli placed some plant buds on detectors with photomultiplier tubes for measurement and observed ultra weak luminescence (UWL) phenomenon^10^. Subsequently, a large number of experiments have proved that UWL is a common life phenomenon that is related to biological or physiological activities as well as information exchange or energy transmission processes^11–13^. Up to now, detection technology based on UWL has been applied in various grain quality analysis fields and obtained fruitful achievements, including hidden insects detection^14^, seedling germination testing^15^, wheat fresh degree classification^16^, and so on. Duan et al.^17^ applied the permutation entropy algorithm to analyze the UWL signals between the healthy and the infected wheat and then used a back propagation (BP) neural network to establish the detection model, and the classification accuracy reached 90%. Similarly, this paper took advantage of UWL signals of healthy wheat samples and moldy wheat samples, using multiscale approximate entropy as the main classification features, and then resorted to support vector machine (SVM) to establish the detection model.

## Materials and methods

### Materials

#### Wheat samples

**Figure 1:**
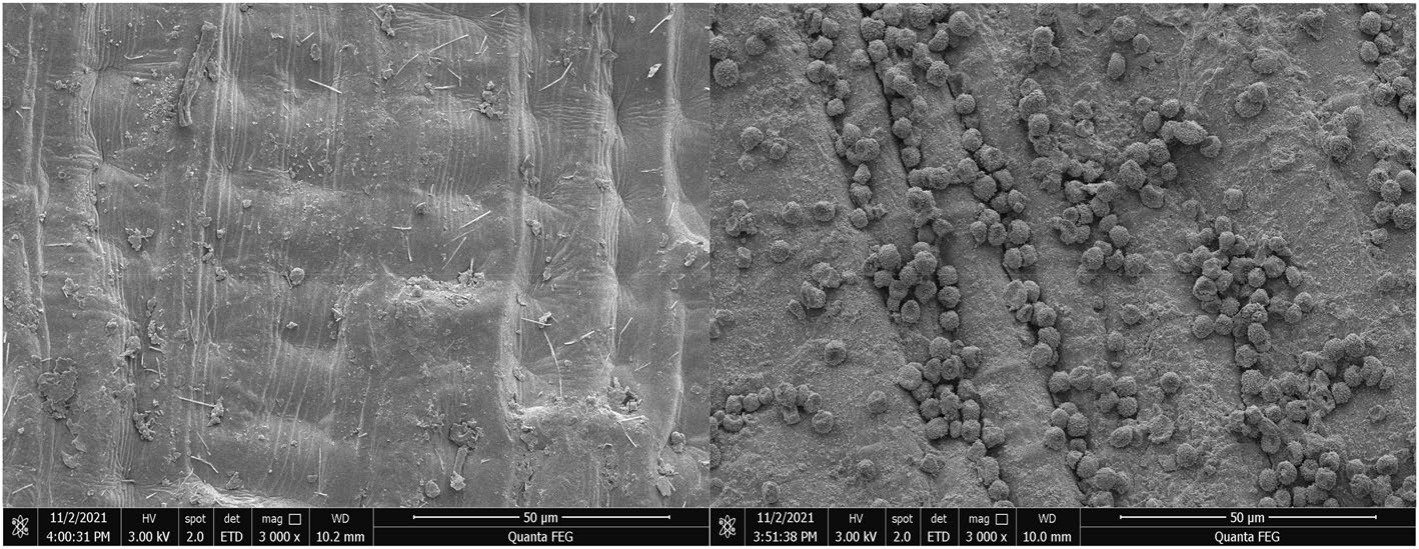
Comparison images between the healthy and the moldy wheat carrying 50% AFB1 under the electron microscope.

Original wheat sample in the year of 2019 was offered by the Yuda grain barn, Zhumadian city, Henan Province, China. Before performing the subsequent experiments, some pretreatments, including picking out foreign materials and damaged kernels, washing wheat kernels three times using distilled water, drying the sample to the degree of moisture at 12.5% using electric blast drying oven were of necessity. Subsequently, the original wheat sample was divided into two parts: one part was selected as the healthy sample, and the other part was sent to the College of Biological Engineering to cultivate the moldy sample with 50% *Aspergillus flavus*. The moldy wheat sample was made by the following steps: firstly, the spores of *Aspergillus flavus* were inoculated on the Potato Dextrose Agar (PDA) medium plate, and cultured in the Constant Temperature and Humidity Incubator (Its type: ZSXD-A1430) manufactured by Nanjing Qianxi Instrument and Equipment Co., LTD at the temperature of 28 °C for 6 days, and then washed the spores of *Aspergillus flavus* with a small amount of aseptic water. Secondly, the suspension of *Aspergillus flavus* spores with concentration of 10^5 cfu/ml was prepared by counting hemocytometer. Furthermore, *Aspergillus flavus* spore suspension was inoculated on wheat surfaces and mixed evenly according to 5% ratio (weight/weight), and then placed into the incubator at the temperature of 28 °C to culture 30 days. Finally, once the moldy wheat was available, we mixed the moldy wheat with healthy wheat evenly according to 1:1 proportion in order to obtain the final moldy wheat sample, and then placed them into electric blast drying oven make the moldy sample at the same moisture degree as the healthy sample. Figure 1 shows the surface images of the healthy and the moldy wheat under the electron microscope. To the healthy wheat sample, we prepared 240 subsamples and the weight of each subsample is (20.00 ± 0.01) g. Half (120) subsamples were used as the training set (experimental set), and the other half were prepared for the testing set. To the moldy wheat sample, we prepared the subsamples according to the same partitions as the healthy ones. Meanwhile, protective measures were taken during this process due to the strong poisonous of AFB1. All the subsamples were stored in the fridge at the temperature of 4 °C for the sake of minimizing influences caused by external environment.

#### Equipment

**Figure 2:**
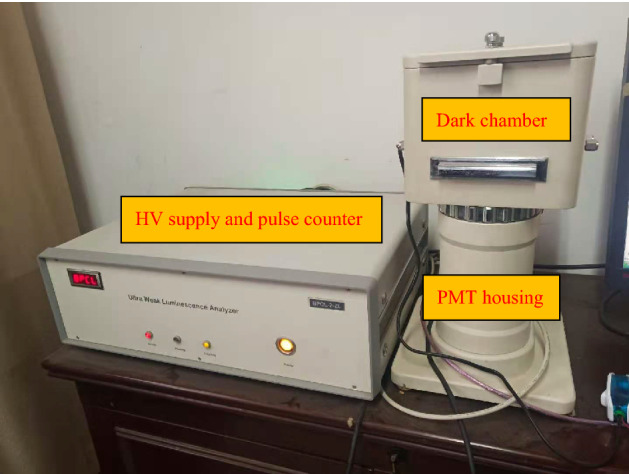
Ultra weak luminescence measurement instrument used in the experiment.

The BPCL-2-ZL, manufactured by Beijing Jianxin Lituo Technology Co., Ltd., was used to measure the photon signals of healthy and moldy wheat subsamples. Figure 2 shows the whole measurement instrument, which consists of two parts: (1) detection chamber, where subsample is placed; (2) photon analyzer, which consists of a photon counting and optical hi-voltage converter device.

The parameters of photon analyzer are as follows:

- average background noise: 26counts/s
- testing spectral range: 300–650 nm
- fastest sampling rate: 0.1 ms
- peak voltage output: 1500 V
- working conditions: voltage (220 V, 50 Hz), temperature (5–40 °C), relative humidity (20–80%).

In the following tests, testing temperature is set as (20.0 ± 0.5) °C and high voltage is 1030 V.

### Methods

Above all, all the following experiments on wheat samples were carried out according to the institutional guidelines. The whole detection process consisted of two stages. First stage was selecting the suitable environmental measurement parameters for measuring UWL signals of the subsamples. Since the UWL signals of two types of wheat samples were easy to be influenced by the surrounding factors, finding the optimum measurement environment was of priority. Consequently, all the measurements were conducted under the same conditions to minimize the environmental influences. After many measurements, we set the environmental temperature as (20 ± 1 °C), humidity (25 ± 6%), and measuring time (8:00 am–18:30 pm). The other stage was choosing the right experimental parameters. We took out the subsamples from the fridge two hours in advance before testing and each subsample was placed in a dark space for 30 min in order to decrease the interference from ambient stray light. Since the UWL signal of wheat subsample was not strong enough, the sampling interval was set to 10 s so as to collect enough counts of photons. In order to better reflect the properties of the UWL signals of two types of wheat samples, the total sampling time was extended over 15,000 s. Furthermore, we reserved the rest of samples into a fridge at the temperature of 4 °C while finishing measurement on that very day so as to minimize the influences caused by external factors.

## Results

### Photon data analysis

**Figure 3:**
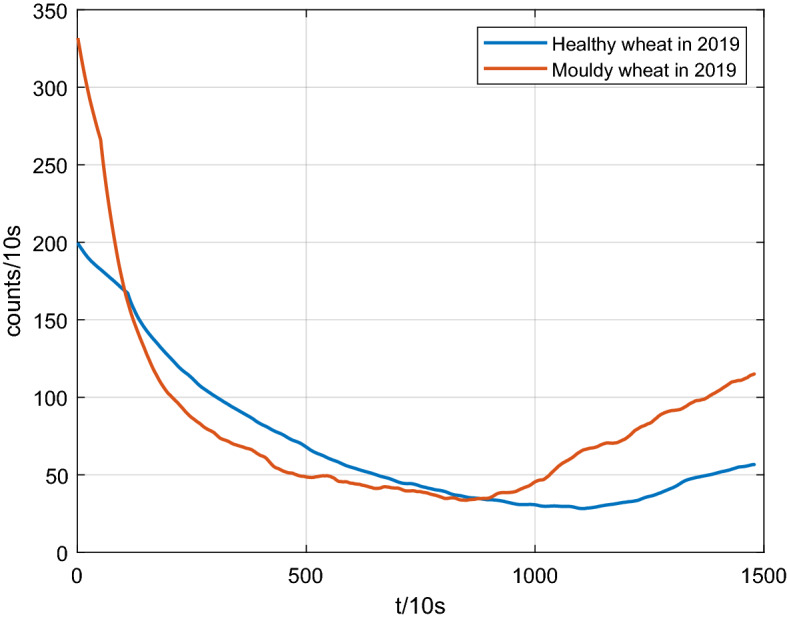
Average UWL data of the healthy and the moldy wheat subsamples.

Before measuring the UWL signals of two types of wheat subsamples, we took a “blank” measurement in order to obtain the average background noise of analyzer. After several measurements, we calculated the average background noise of the analyzer is 238 counts/10 s, and then we adopted the subtraction background mode in the following measurements, namely, the UWL signals of all wheat subsamples we measured were subtracted this average background noise from each initial UWL signal. The UWL signals of the healthy and the moldy wheat subsamples were measured separately according to the above-mentioned processes. Subsequently, we calculated the average value of UWL signals of each type of wheat subsamples, and the results were shown in Fig. 3. Seen from the Fig. 3, it was obviously observed that the previous segment of UWL signal (within 10,000 s) exhibited a delayed luminescence characteristics. Thus, in order to obtain a more accurate data of UWL, we took advantage of the posterior segment of UWL signal from 10,010 to 15,000 s. Table 1 showed three statistic parameters of UWL data of two types of wheat, and they were mean, variance and standard deviation successively. Seen from the Table 1, the mean of UWL signal of moldy wheat was larger than that of healthy wheat, which illustrated that the *Aspergillus* fungi carried by moldy wheat kernels had stronger metabolism and respiration functions. Simultaneously, the stronger UWL signal of moldy wheat provided a convincing explanation, which coincided with a physiological regularity that the higher grade of an organism is, the stronger UWL signal it radiates.

**Table 1:** Three statistic parameters of UWL data of two types of wheat subsamples.

Healthy wheat kernels in 2019		Moldy wheat kernels in 2019
Mean	38.92	82.24
Variance	636.61	1033.83
Standard deviation	25.23	32.15

**Figure 4:**
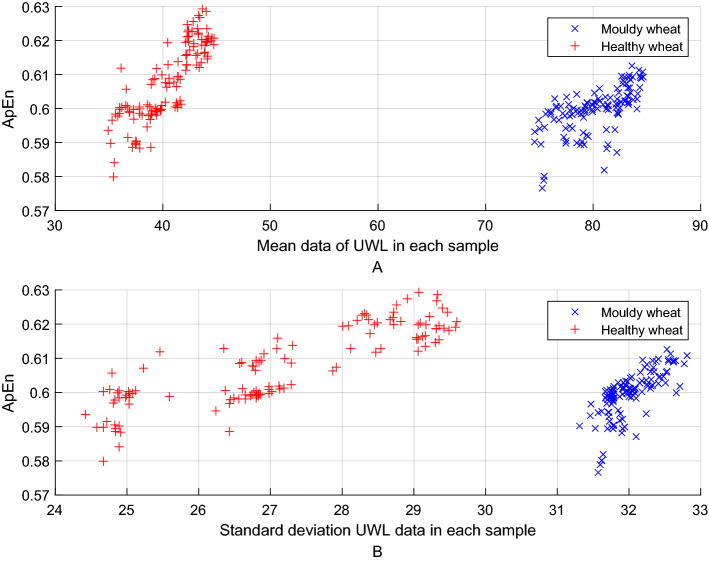
ApEn value of the healthy and the moldy wheat under different statistical parameters.

To effectively classify the healthy and the moldy wheat based on UWL signals, we take advantage of the approximate entropy (ApEn) as one of the classification features. Due to the strong relativity between variance and standard deviation, we finally choose the mean, standard deviation and ApEn value as the main classification features, which is shown in Fig. 4. Subsequently, a detailed introduction about multiscale approximate entropy (MApEn) algorithm can be seen in the following part.

### Multiscale approximate entropy

ApEn algorithm was proposed by the scholar Pincus for the sake of measuring the characteristics of random series^18^. The more complex an initial time series is, the larger its corresponding ApEn value shows. The ApEn algorithm is suitable to analyze the UWL signals of wheat because of its robust performance. Two striking advantages of the ApEn algorithm are its lower dependency on the length of the initial time series and strong resistance to the noise existed in the original data. Since the computation process of ApEn was quite complex, Bo et al.^19^ proposed a type of fast ApEn algorithm that shortened the computing time by nearly 5 times and the main steps were as follows:

First step: the distance matrix $D(N \times N)$ of the initial $N$ points time sequence is computed, and the element in the $i{\text{th}}$ row and $j{\text{th}}$ column is denoted as $d_{ij}$. The rules of calculating $d_{ij}$ are based on the following formula:

1 $$d_{ij} = \left\{ {\begin{array}{*{20}c} {\begin{array}{*{20}c} 1 & {|x(i) - x(j)| < r} \\ \end{array} } \\ {\begin{array}{*{20}c} 0 & {|x(i) - x(j)| \ge r} \\ \end{array} } \\ \end{array} \begin{array}{*{20}c} {} & {i = 1\sim N;j = 1\sim N;i \ne j} \\ \end{array} } \right.$$

Second step: assuming the dimension $m = 2$, we can easily obtain the values of $C_{i}^{2} (r)$ and $C_{i}^{3} (r)$ using Eqs. (2) and (3).

2 $$C_{i}^{2} (r) = \sum\limits_{j = 1}^{N - 1} {d_{ij} \cap d_{(i + 1)(j + 1)} }$$

3 $$C_{i}^{3} (r) = \sum\limits_{j = 1}^{N - 2} {d_{ij} \cap d_{(i + 1)(j + 1)} } \cap d_{(i + 2)(j + 2)}$$

Third step: calculate the logarithm of $C_{i}^{m} (r)$, and then obtain its mean using Eq. (4). Here, the mean is labeled as $H^{m} (r)$.

4 $$H^{m} (r) = \frac{1}{N - m + 1}\sum\limits_{i = 1}^{N - m + 1} {\ln C_{i}^{m} (r)}$$

By increasing the dimension from $m$ to $m + 1$ and repeating  steps 2–4, and then $H^{m + 1} (r)$ can be obtained.

Fourth step: since $N$ is finite, the ApEn can be described as:

5 $$ApEn\left( {m,r,N} \right) = H^{m} \left( r \right) - H^{(m + 1)} \left( r \right)$$

In order to improve the robust performance of ApEn algorithm, the multiscale approximate entropy (MApEn) algorithm has been introduced in this paper. Interestingly, compared with only one feature obtained by ApEn algorithm, MApEn algorithm are able to offer a cluster of classification features to train the detection model. The detailed computation steps of the MApEn algorithm are as follows^20^:

1. Assume the initial time series is $X = \{ x(i), \, i = 1,2, \ldots ,N\}$, and its length is $N$.
2. Construct a coarse time series $\{ z^{(\tau )} \}$, where $\tau$ represents the scale factor, and then the scaling time series can be expressed as:
  6 $$z^{\tau } (j) = \frac{1}{\tau }\sum\limits_{i = (j - 1)\tau + 1}^{j\tau } {x(i)} \;\;1 \le j \le N/\tau$$

Equation (6) is the same as the original sequence provided that the scale factor $\tau { = 1}$. Furthermore, each coarse-graining series can be regarded as evenly dividing the original series, and the length of each segment is $\tau$.

**Figure 5:**
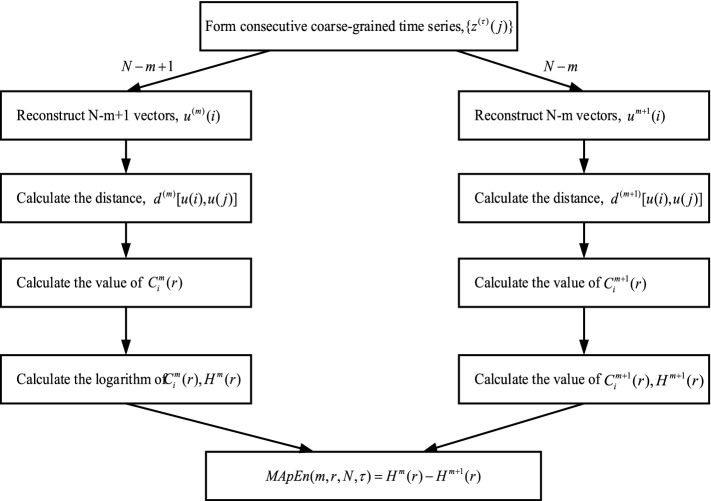
Flowchart of multiscale approximate entropy algorithm.

By combining multiscales with approximate entropy, MApEn algorithm is able to characterize the nonlinear information of series more effectively. Figure 5 shows the detailed flowchart of the MApEn algorithm.

### The performance of MApEn algorithm

#### Parameter selection

There exist four parameters in MApEn algorithm: the length of the initial UWL signal $N$, the dimension of the pattern vector $m$, the similar tolerance threshold value $r$, and the time scale factor $\tau$. To the ApEn algorithm, selecting the right parameters is of extreme importance.

**Figure 6:**
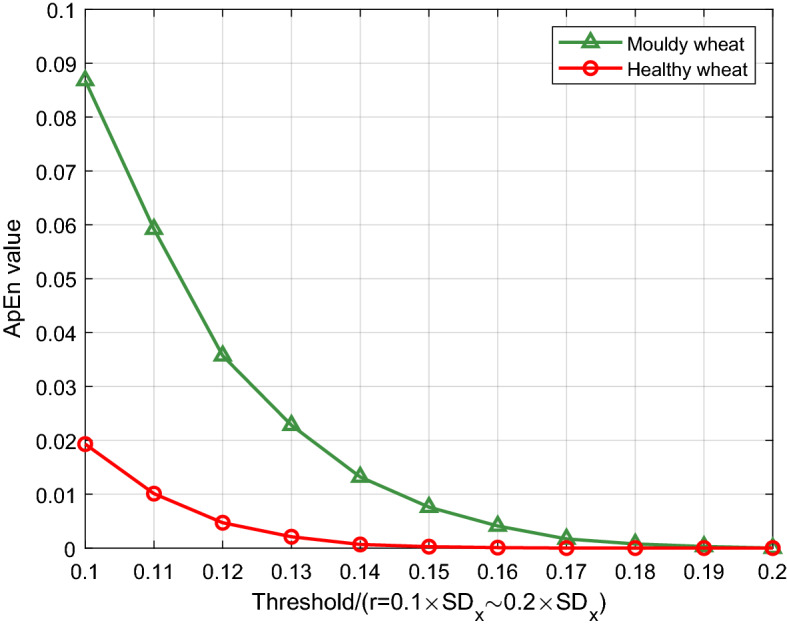
ApEn values of UWL signals of the healthy and the moldy wheat with different tolerance thresholds.

After several tests, we finally selected $N = 500, \, m = 2, \, r = 0.12 \times SD_{x}$ as the experimental parameters, where $SD_{x}$ represented the standard deviation of initial time series. The ApEn values of the UWL signals of the two types of wheat at different tolerance thresholds were simulated by Matlab 2018a, and the results were shown in Fig. 6. As shown in Fig. 6, the ApEn values of the two types of wheat varied depending on different tolerance thresholds $r$. Especially, while $r$ value was from $0.1 \times SD_{x}$ to $0.15 \times SD_{x}$, the differences of ApEn values between the healthy and the moldy wheat were obvious. In addition, another conclusion from the experimental results is that the lager ApEn value of the moldy wheat reflects that the activities of *Aspergillus* fungi are more irregular and intensive, and thus, the ApEn value can be used as an effective index to feature them.

## Discussions

### Performance analysis of the MApEn algorithm

**Figure 7:**
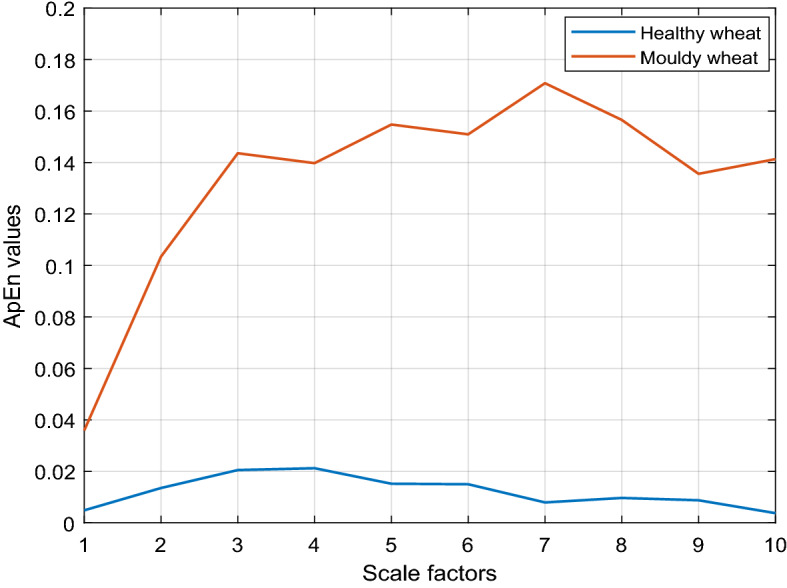
MApEn values of UWL signals of two types of wheat with different scale factors.

The ApEn algorithm only offers one classification feature; therefore, in order to overcome this shortcoming and obtain more classification features, the MApEn algorithm is introduced in this paper. To the ApEn algorithm, the parameters $N = 500, \, m = 2, \, r = 0.12 \times STD$ are finally chosen and simulated through experiments. Besides three parameters mentioned above, the scale factor $\tau$ is a decisive factor to the performance of the MApEn algorithm. Due to the limited length of the initial time series, $\tau$ is usually taken a value from 2 to 10. The MApEn values of UWL signals of two types of wheat with different scale factors are shown in Fig. 7.

Shown by Fig. 7, the following conclusions can be obtained:

1. Compared with ApEn values (when $\tau = 1$), the MApEn values (when $\tau \ge 2$) of UWL signals of the two types of wheat sample obviously enlarge their differences;
2. MApEn algorithm can provide several classification features at the same time that can be used to feature the original UWL signal rather than only one feature offered by ApEn algorithm.

### Bi-classification and performance assessment by SVM

To solve the classification problem between healthy and moldy wheat, SVM classifier has been introduced in this paper. SVM, proposed by Cortes and Vapnik^21^, is a type of linear classifier based on classification boundaries. Computationally, the striking points of SVM classifier are how to choose kernel functions, and the kernel functions map the nonlinear transformation of the input feature space from a lower-dimensional space into a higher-dimensional space. In other words, this problem can be considered as an optimization problem in which we seek to help the kernel function to find out the optimal plane, by which we can carry out linear classification through a nonlinear transformation^22^. Even if the training dataset are not large enough, SVM classifier can achieve a good classification performance^23^. Up to now, the SVM has become one of most widely used classifiers, which has been applied in various classification research fields^24,25^.

**Figure 8:**
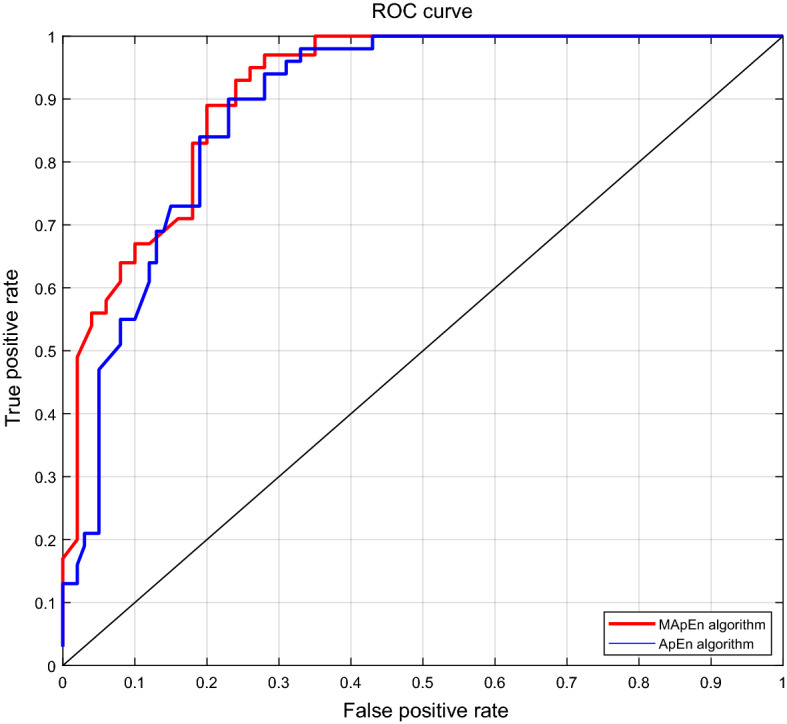
The ROC curves of bi-classification models.

To ApEn algorithm, we extracted two statistics parameters (mean and standard deviation) in Table 1 and ApEn value as the feature vector to represent the UWL signal of each wheat subsample, among which 120 groups were selected as the training group and the other 120 groups were used as the testing group. To MApEn algorithm, we extracted the same statistics parameters but using MApEn values instead of ApEn value to construct the feature vector. Subsequently, we resorted to SVM classifier to establish the finial classification model, and the main parameters of the SVM were set as follows. The type of kernel function was a radial basis function, and the error value that terminated the iteration was 0.001. The receiver operating characteristic (ROC) curve was shown in Fig. 8, where the blue and the red curve represented the classification performance of the ApEn and the MApEn algorithm respectively.

Based on the ROC curves in Fig. 8, Tables 2 and 3 gave four indices to assess the classification performance, where AUC, S.E., C.I., and PA represented the area under the curve, the standard error of the area, the confidence interval and the performance of the classifier separately. Through comparing the indices in Table 2 with those in Table 3, we observed that the classification accuracy rate based on MApEn algorithm was improved obviously and the standard error was decreased by introducing the MApEn algorithm. The experimental results validated that the MApEn values were able to act as a cluster of main classification features to classify the healthy wheat and the moldy wheat subsamples infected by 50% AFB1.

**Table 2:** Classification performance indices using ApEn value as the main classification feature.

AUC	S.E	95% CI	PA
0.8724	0.0233	[0.8276–0.918]	Good

**Table 3:** Classification performance indices using MApEn values as the main classification features.

AUC	S.E	95% CI	PA
0.8881	0.0218	[0.8453–0.9309]	Good

## Conclusions

UWL signals of wheat kernels under different conditions can reflect their inner physiological and pathological changes; therefore, it offers us a new thought to assess their quality states based on the UWL signals.

MApEn algorithm was introduced to feature the UWL signals in this paper. Subsequently, we established the classification model based on the SVM classifier. The experimental results showed that the MApEn algorithm was efficient and practical in featuring UWL signals. One main deficiency was that we only established a bi-classification model in this paper due to the limited training dataset. Furthermore, since detecting moldy wheat kernels is a continuous process during the storage period, establishing a multi-classification model to classify the degree of moldy wheat is of extreme significance, which requires further research to improve the detection performance of the established model.

 As UWL signal of wheat subsample is so sensitive to environmental factors, further studies and experiments seeking to minimize these influences and improving the efficiency of measuring UWL signal need to be conducted. Moreover, extensive research beyond the scope of this paper needs to be carried out to set up a detection model based on the delayed luminescence signals of wheat subsamples.

## Data Availability

The datasets used and/or analysed during the current study available from the corresponding author on reasonable request.
